# Immune Responses of the Black Soldier Fly *Hermetia illucens* (L.) (Diptera: Stratiomyidae) Reared on Catering Waste

**DOI:** 10.3390/life13010213

**Published:** 2023-01-11

**Authors:** Valentina Candian, Marco Meneguz, Rosemarie Tedeschi

**Affiliations:** 1 Dipartimento di Scienze Agrarie, Forestali e Alimentari (DISAFA), University of Torino, Largo P. Braccini 2, 10095 Grugliasco (TO), Italy; 2 BEF Biosystems, Via Tancredi Canonico 18/c, 10156 Torino (TO), Italy

**Keywords:** circular economy, antimicrobial peptides, defensin, cecropin, hemolymph, diet

## Abstract

The black soldier fly (BSF), *Hermetia illucens* L. (Diptera: Stratiomyidae), has a great bioconversion potential and ability to develop on diverse substrates. Although the use of catering waste and food by-products containing meat and fish would reduce the footprint of the insect sector, to date, in Europe, their use is still facing legal obstacles for insects as food and feed. Since a major request from the EU insect sector is to diversify the spectrum of allowed substrates, and considering that variations in diet composition could influence insect immune responses, we evaluated the impact of different catering wastes on BSF prepupae immunity. Insects were reared on five diets: one based on feed for laying hens and four based on catering waste containing (i) fruits and vegetables; (ii) fruits, vegetables and bread; (iii) fruit, vegetables, bread and dairy products; (iv) fruits, vegetables, bread, meat and fish. The gene expression of two antimicrobial peptides (AMPs), one defensin and one cecropin, was assessed. Moreover, the hemolymph inhibitory activity against *Escherichia coli* DH5α and *Micrococcus yunnanensis* HI55 was evaluated using diffusion assays in solid media. The up-regulation of both AMPs’ encoding genes was observed in insects fed a bread-added and dairy product-added diet. All hemolymph samples showed inhibitory activity against both bacteria, affecting the colony size and number. The obtained results show how catering waste positively influences the *H. illucens* immune system. The possibility of modulating AMP expression levels through the diet opens up new perspectives in the management of insect health in mass rearings.

## 1. Introduction

It has been estimated that around 14 percent of the world’s food is lost after harvest and before it reaches the shops, and a further 17 percent becomes waste in retail and by consumers [1]. In most developed and developing countries, food waste is the greatest component of municipal solid waste [2]. This significant amount of food that is produced, but not eaten, has remarkable impacts on the environment, society and the economy. Suffice it to think that 8–10% of global greenhouse gas emissions are associated with food that is not consumed [1,3]. Even though waste minimization should be the main key strategy to reduce the consequences of this issue, the search for new solutions for the valorization of these biomasses, that are still rich in carbohydrates, proteins and lipids, is needed in a circular economy context. Different studies investigate the potential for food waste recovery and most of these materials are currently recycled by feeding to livestock, anaerobic digestion, composting and creating bio-energy and natural fertilizers [4,5].

More recently, the insect-based bioconversion represents an economically viable solution for food waste management [6,7,8,9,10]; according to IPIFF [11], up to one-third of the food waste generated today could be suitable for insect farming. This method can efficiently turn many tons of food waste into valuable products, including human food, animal feed, fertilizers and secondary industrial compounds [12]. To date, only a few insect species are commercially proposed for this kind of insect-based bioconversion, and the black soldier fly (BSF) *Hermetia illucens* L. (Diptera: Stratiomyidae) is the most promising one [13,14,15,16]. BSF larvae have the ability to rapidly consume large amounts of a wide variety of organic wastes, including animal manure, fecal sludge, meat and kitchen waste, and for this they can successfully be used to reduce organic waste and produce valuable nutritious prepupae as animal feed [17]. In addition to being robust decomposers, BSF larvae contain significant amounts of protein and lipids and have gained substantial interest as a dietary supplement to feed chickens, swine and fish [13]. In particular, the replacing of the dietary high-value and unsustainable fish meal with the less expensive BSF larvae meal is a highly topical subject for the sustainable development of the aquaculture sector with several successful examples [18,19,20,21,22]. Moreover, dietary BSF has been shown to enhance gut health, gut microbiome, immunity and fish welfare at certain levels of inclusion [23].

However, there are still some challenges in rearing insects for feed on food waste that must be taken into account. Physical, chemical and microbiological characteristics of these kinds of substrates, their stability and the occurrence of non-organic contaminants, such as plastics or aluminum foils, should be considered [9]. Moreover, variations in the diet nutrient profile, and, more specifically, the protein/carbohydrate ratio content of the food waste-based diet influence both insect-growing performances and their immune responses. Indeed, the substrate availability and its protein/carbohydrate ratio are typically used to evaluate the diet impact on insect immunity [24,25]. Under nutritional deficiency conditions, insects present difficulties in implementing immune responses [26], and a protein/carbohydrate ratio in favor of the proteins usually determines a greater production of antimicrobial peptides (AMPs), a greater number of hemocytes present in the hemolymph and more frequent encapsulation responses [26,27].

The possibility of modulating both the cellular and humoral immune responses of an insect through the diet has only recently been highlighted [27,28,29,30,31]. The effect of the diet on the expression of AMPs, a key component of the insect’s humoral responses, in BSF has been recorded providing diets containing high microbial loads or supplemented with cellulose, chitin, lignin, brewer’s grains, protein, and sunflower oil [25,31]. Variation in the diet of *Hyphantria cunea* Drury [Lepidoptera: Erebidae] results in an increase in the hemocytes’ number in the hemolymph [32]. Even if this early evidence demonstrates the possibility of modulating the insect’s immune system through the diet, there is still a lack of research on this topic. Therefore, it is necessary to evaluate thoroughly the largest number of suitable substrates in order to preserve insect health in mass rearing.

From a circular economy perspective, it is interesting to assess the feasibility of using a waste-based diet for insect mass rearing by recovering material easily available on the territory, such as catering waste. To date, in Europe, the use of catering waste (including canteen and household waste) and food by-products containing meat and fish is only admitted for some insect rearings, while it is still facing legal obstacles for insects farmed as food and feed. Furthermore, widening the spectrum of allowed substrates (as requested from the EU Insect Sector) will play a key role in enhancing the circularity of insect production, thus helping European insect farms to reach their full potential. In this scenario, considering also that variations in diet composition could influence insect immune responses, we evaluated the impact of different catering waste on BSF prepupae immunity. In particular, we tested the impact of different food waste on the expression of two genes coding for AMPs (one defensin and one cecropin). The inhibitory activity of the hemolymph, extracted from prepupae reared on the different food waste, was also evaluated against one Gram-negative and one Gram-positive bacterium.

## 2. Materials and Methods

### 2.1. Insect Rearing

The BSF was reared at the BEF Biosystems s.r.l. production facility located in Casalnoceto (Italy) in a climate-controlled chamber (T: 27 °C; RH: 70%, 14:10 h L:D photoperiod). Groups of eggs (1 g each) were positioned on a net placed above plastic containers (10 × 17.5 × 7 cm) filled with 400 g of a Gainesville diet (30% alfalfa, 50% wheat bran, 20% cornmeal, and a 70% moisture content [33]). After six days, all larvae were sieved and then weighed individually.

In order to evaluate the growing performances of the insects, the substrate reduction and the effect of the diet on the AMPs’ expression level and on the hemolymph inhibitory activity, different diets were compared. In total, 5 diets were used: (i) NaturOvo^®^ Pellet complete feed for laying hens (NatOvo) [Cargill S.r.l., Fiorenzuola d’Arda, Italy] as the control diet; (ii) a diet composed of 60% fruits (pineapple, apple and pear) and 40% vegetables (lettuce, broccoli, fennel, spinach, carrots and tomato) (FV); (iii) a diet composed of 40% fruits, 40% vegetables and 20% bread (FVB); (iv) a diet composed of 40% fruits, 40% vegetables, 10% bread and 10% dairy products (parmesan cheese and fresh dairy products) (FVBD); (v) a diet composed of 30% fruits, 30% vegetables, 10% bread and 30% fish and chicken meat (FVBM). Catering waste used as a rearing substrate were recovered in five locations in south-western Lombardy in northern Italy (Pavia). Specifically, catering waste from two retirement homes, two restaurants and a canteen were collected. In addition, other specimens of BSF, of African origin, were reared on NatOvo.

For each diet and BSF population, 3 repetitions of 2000 six-day-old larvae each were set up. The diet was provided in one solution at the time of setting up the experimental trial. Larvae were reared in a plastic container (30 × 40 × 15 cm) and fed with 2 kg of the different tested diets in a climate-controlled chamber (T: 28.5 ± 1.5 °C; RH: 70%, 14:10 h L:D photoperiod). After 7 days of permanence on the tested diet, the necessary time in order to reach the economically suitable stage, different larval production parameters and growing performance were assessed. For each repetition, the individual final weight of 300 larvae, the average daily gain (ADG) of groups of 50 larvae, the survival rate, the final larval biomass produced and the substrate reduction were evaluated on BEF Biosystems larvae reared on NatOvo, FV, FVB, FVBD and FVBM. The ADG, the survival rate and the substrate reduction were calculated according to the formulas below:

ADG = (final weight − initial weight)/number of days on diet(1)

Survival rate = (final no. of larvae/initial no. of larvae) × 100(2)

Substrate reduction = (initial substrate weight/final substrate weight) × 100(3)

In order to assess the effect of the diet on the AMPs’ expression level and on the hemolymph inhibitory activity, all experiments were stopped when 10% of the larvae reached the prepupal stage. Prepupae were sieved and washed in diethylpyrocarbonate (DEPC) water [Merck KGaA, Darmstadt, Germany], 75% ethanol in DEPC water, and DEPC water for 2, 1 and 2 min, respectively, with the aim of removing any diet residues and any other possible contaminant present in it (e.g., insect frass, bacteria). For each diet and each population, half of the collected individuals were stored in RNAlater^®^ [Merck KGaA, Darmstadt, Germany] at −20 °C until total RNA extraction, while the other part was immediately subjected to hemolymph extraction.

### 2.2. Gene Expression Analysis

Total RNA extraction was performed following the TRI Reagent^®^ protocol [Merck KGaA, Darmstadt, Germany]. Briefly, BSF prepupae were grounded to a fine powder under liquid nitrogen and lysed in 600 µL of TRI Reagent^®^; then, samples were incubated at room temperature for 5 min. Cleared lysate solutions were obtained by centrifugation, and subsequently 60 µL of BCP (1-Bromo-3-chloropropane) [Merck KGaA, Darmstadt, Germany] was added, and samples were incubated at room temperature for 15 min. After centrifugation, 300 µL of isopropanol [Merck KGaA, Darmstadt, Germany] was added and incubated at room temperature for 10 min prior centrifugation. Finally, samples were washed once with 75% ethanol and resuspended in 50 µL nuclease-free water. For each diet and each population, total RNA was extracted from 20 specimens. RNA quality and concentration were assessed with an ND-1000 spectrophotometer [NanoDrop Technologies, Wilmington, DE, USA]. Subsequently, 0.8–1 µg of RNA was used for cDNA synthesis by the iScriptTM cDNA synthesis kit (Bio-Rad, Hercules, CA, USA). Then, the cDNA was diluted (1:10) and a quantitative real-time PCR (qPCR) was used in order to assess the AMPs’ coding gene expression levels.

#### Quantitative Real-Time PCR

Reactions were performed with the SensiMixTM SYBR^®^ No-Rox kit [Bioline Meridian Bioscience, London, UK] using primers targeting the defensin (Forward: 5′-TCGTCCCATGGCAATACAAT-3′ and Reverse 5′-TAGTGGAGCAGCATTATCGGG-3′) and cecropin coding genes (Forward: 5′-GGTCAAAGCGAAGCTGGTT-3′ and Reverse 5′-TGCCAGAACATTGGCTCCTT-3) designed by Candian et al. [31] on AMP protein sequences predicted by the transcriptome analysis by Vogel et al. [25]. Actin coding gene (Forward: 5′-TTCGAGCAGGAAATGGCCAC-3′ and Reverse 5′-TTGGAAGAGAGCCTCTGGAC-3′) was used as reference gene [34].

Analyses were conducted in clear HardShell^®^ Low-Profile 96-Well PCR Plates (Bio-Rad, Hercules, CA, USA) with a 50 µL mixture containing 25 µL of SYBER^®^ Green, 0.5 µL of each primer (25 µM), 5 µL of cDNA sample and 19 µL of sterile H_2_O, sealed with adhesive Microseal^®^ PCR Plate Sealing Film (Bio-Rad, Hercules, CA, USA); samples were analyzed in triplicate. The analysis was performed on a CFX ConnectTM Real-Time PCR Detection System (Bio-Rad, Hercules, CA, USA), applying the following thermic protocol: 95 °C for 10 min, followed by 40 cycles of 95 °C for 15 s, 58.5 °C for 15 s and 72 °C for 15 s. A final step for the melting curve analysis from 58.5 to 95 °C, measuring fluorescence every 0.5 °C, was added. Results were analyzed using the CFX ManagerTM Software (Bio-Rad, Hercules, CA, USA) for Ct determination. Relative quantification of the target genes was calculated using the 2^−ΔΔCt^ method [35] and expressed as a fold change.

### 2.3. Hemolymph Inhibitory Activity

In order to extract the hemolymph, the protocol designed by Tabunoki et al. [36] and slightly modified as described in Candian et al. [31] was used. Briefly, the insect’s thorax was gently injured with a scalpel, then all steps were performed maintaining the samples directly on ice. Specimens were centrifuged individually by means of a refrigerated centrifuge Z 326 K^®^ [Hermle Labortechnik GmbH, Wehingen, Germany] for 5 min at 590× *g* at 4 °C. The obtained supernatant was subsequently centrifuged for 10 min at 21,380× *g* at 4 °C in order to precipitate the hemocytes and any impurities previously collected. The new supernatant was collected and stored at −20 °C until further analyses. For each diet and for each population, 60 prepupae were used for the hemolymph extraction. For each diet/population, the hemolymph totally extracted was combined to obtain the pooled samples and stored at −20 °C until further analyses.

The hemolymph inhibitory activity was tested against one Gram-negative bacterium, *Escherichia coli* DH5α, and one Gram-positive bacterium isolated from BSF [37], *Micrococcus yunnanensis* HI55, in diffusion assays in solid media. Briefly, bacteria were grown overnight in 5 mL of LB broth [Merck KGaA, Darmstadt, Germany] at 30 °C in a thermostatic dome shaker VDRL 711/CT^®^ [Asal srl, Cernusco sul Naviglio, Italy]. The final concentration of the bacteria inoculum was adjusted to 7.27 × 10^7^ CFU mL^−1^ of *E. coli* DH5α and 1.30 × 10^7^ CFU mL^−1^ of *M. yunnanensis* HI55 using phosphate-buffered saline (PBS, 137 mM NaCl, 2.7 mM KCl, 10 mM Na_2_HPO_4_/KH_2_PO_4_, pH 7.4) [Merck KGaA, Darmstadt, Germany]. Petri dishes (Ø 6 cm) [Sarstedt AG & Co., Nümbrecht, Germany], containing 13 mL of LB Broth with agar (Lennox) (43% agar) [Merck KGaA, Darmstadt, Germany], were inoculated with 50 µL of the bacteria solution. Following the complete absorption of the bacteria inoculum, 10 µL of hemolymph was applied directly in the center of the agar surface. For each diet, population and bacteria inoculum, 3 repetitions were set up. Furthermore, for each bacterium, 3 inoculated plates without hemolymph were used as the untreated control while another 3 inoculated plates, without hemolymph but added with a disc of bibulous paper (Ø 6 mm) [Biosigma SpA, Cona, Italy] soaked with 26 µL of ampicillin (50 mg mL^−1^) and placed in the center of the plate, were used as the antibiotic-treated control. Plates inoculated with *E. coli* DH5α were incubated at 37 °C, while the ones inoculated with *M*. *yunnanensis* HI55 were incubated at 30 °C. The hemolymph inhibitory activity was observed after 24 h and 48 h of incubation.

### 2.4. Statistical Analysis

Statistical analyses were performed with SPSS Statistics 28 (IBM Corp. Released 2017, Armonk, NY, USA). Larval final weight, ADG and final larval biomass were analyzed as a number, while the survival rate and substrate reduction percentage data were analyzed following arcsin square root transformation. Gene expression data were subjected to a logarithmic (log10) transformation for normality before the statistical analysis. All data were checked for homogeneity of variance (Levene test) and normality (Shapiro–Wilk test) and compared using a one-way analysis of variance (ANOVA); in the case of significant differences, the means were separated by a Tukey’s test. If the assumptions of ANOVA were not met, data were compared using a Kruskal–Wallis test and the means were separated using a Mann–Whitney U test. Outcomes were considered significant at *p* ≤ 0.05.

## 3. Results

### 3.1. BSF Growing Performances and Substrate Reduction

Significant differences among the rearing diets were recorded for the final weight of the individual larva after 7 days of permanence on the tested diet (one-way ANOVA: df = 4, 10; F = 64.238, *p* < 0.001) (Table 1). A heavier weight was recorded in larvae fed a NatOvo and an FVBM diet. Following the same pattern, significant differences among the diets were recorded for the ADG (one-way ANOVA: df = 4, 10; F = 64.147, *p* < 0.001) with the highest value observed in larvae fed a NatOvo and an FVBM diet (Table 1). Significant differences among the rearing diets were recorded for the survival rate (Kruskal–Wallis test: df = 4; H = 12.923, *p* = 0.012) (Table 1). In all the tested diets, the survival rate was higher than 75%. Larvae reared on the FV diet showed the highest survival rate (97.19 ± 0.31), while the lowest was recorded in larvae fed a NatOvo (75.54 ± 2.67) and an FVBM (76.80 ± 1.25) diet. Significant differences among the diets were observed for the final production of larvae biomass (one-way ANOVA: df = 4, 10; F = 39.001, *p* < 0.001) (Table 1). The higher values were recorded with larvae fed a NatOvo (274.50 ± 1.18), an FVBD (263.07 ± 2.00) and an FVBM (271.47 ± 1.75) diet. The substrate reduction ranged from 64.33 ± 2.60% to 86.33 ± 0.38% observed for the FV and the NatOvo diet, respectively (Kruskal–Wallis test: df = 4; H = 13.412, *p* = 0.009) (Table 1).

### 3.2. Gene Expression Analysis

AMP-encoding gene expression levels were modulated differently depending on the insect population and the rearing diet. For the two BSF populations fed a NatOvo diet, a slightly down-regulation of the defensin (fold change: 0.97) and an up-regulation of the cecropin encoding gene (fold change: 1.62) were observed in African specimens compared to the ones reared at the BEF Biosystems and used as the control population.

**Figure 1:**
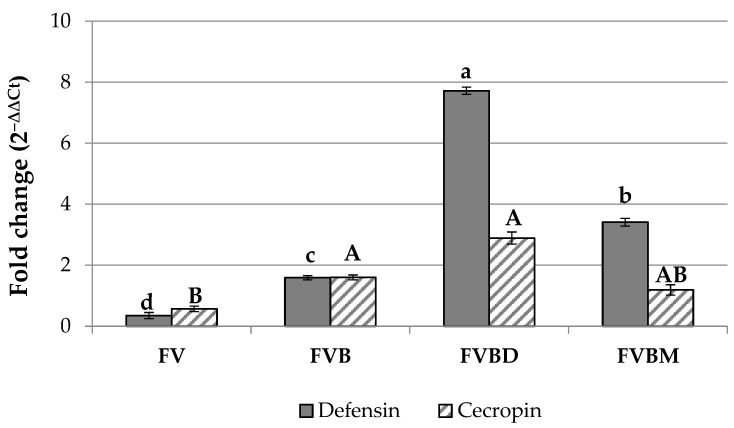
Gene expression (2^−∆∆Ct^) of defensin and cecropin in prepupae reared on different catering waste. Values are reported as average fold change variation (mean ± SE). Samples were normalized against BEF Biosystems BSF prepupae reared on NatOvo. Different letters indicate significantly different values (Kruskal–Wallis test, *p* ≤ 0.05).

Catering waste-based diets caused a variation of defensin and cecropin coding genes’ expression following a distinct pattern according to diet composition. Significant differences among the rearing diets were observed both for defensin (Kruskal–Wallis test: df = 3, H = 39.109, *p* < 0.001) and cecropin (Kruskal–Wallis test: df = 3, H = 14.661, *p* = 0.002) coding genes (Figure 1). A down-regulation of both coding genes was observed in insects fed an FV diet (defensin fold change 0.35, cecropin fold change 0.57). The up-regulation of both AMP-encoding genes was observed in insects fed a bread-added and animal protein-added diet. A fold change of 1.59 and 1.60 for defensin and cecropin was recorded in prepupae reared on an FVB diet, while a fold change of 3.41 and 1.19 for defensin and cecropin, respectively, was observed in prepupae reared on an FVBM diet. The highest transcript levels of both AMP genes were recorded in insects fed an FVBD diet (defensin fold change 7.72; cecropin fold change 2.89).

### 3.3. Hemolyomph Inhibitory Activity

All hemolymph samples showed inhibitory activity against *E. coli* DH5α and *M. yunnanensis* HI55 colonies. Indeed, a generic decrease in the size and number of colonies was observed in all plates inoculated with hemolymph extracted from prepupae fed the tested diets (NatOvo, FV, FVB, FVBD and FVBM).

#### 3.3.1. *Escherichia coli* DH5α

**Figure 2:**
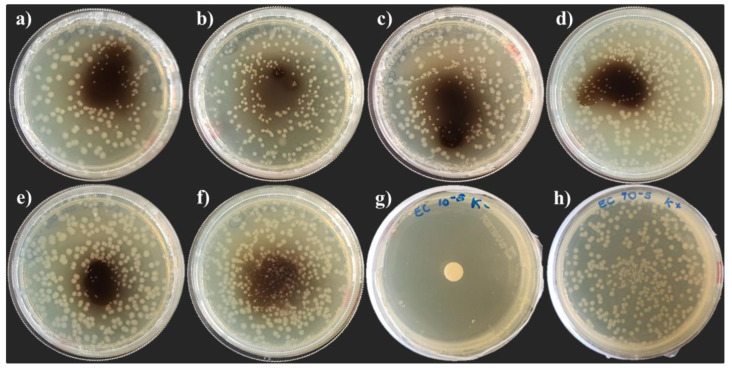
Growth inhibition of *E. coli* DH5α after 24 h of incubation. Radial diffusion assay: (**a**) hemolymph of African prepupae reared on NatOvo, (**b**) hemolymph of BEF Biosystems prepupae reared on NatOvo, (**c**) hemolymph of BEF Biosystems prepupae reared on FV, (**d**) hemolymph of BEF Biosystems prepupae reared on FVB, (**e**) hemolymph of BEF Biosystems prepupae reared on FVBD, (**f**) hemolymph of BEF Biosystems prepupae reared on FVBM, (**g**) antibiotic-treated control, (**h**) untreated control.

After 24 h of incubation, a slight variability of the inhibitory activity of hemolymph extracted from the two BSF populations reared on the NatOvo diet was observed in the development of *E. coli* DH5α colonies (Figure 2). A smaller size of bacteria colonies was recorded in the presence of hemolymph extracted from the *H. illucens* of African origin compared with the one extracted from individuals reared at the BEF Biosystems (Figure 2a,b). A higher inhibitory activity was observed inoculating the plates with hemolymph extracted from individuals fed an FV diet (Figure 2c) and an FVBD diet (Figure 2e) compared with those fed an FVB diet (Figure 2d). Among all the tested diets, the lowest inhibitory activity was recorded with hemolymph extracted from prepupae reared on the FVBM diet (Figure 2f).

**Figure 3:**
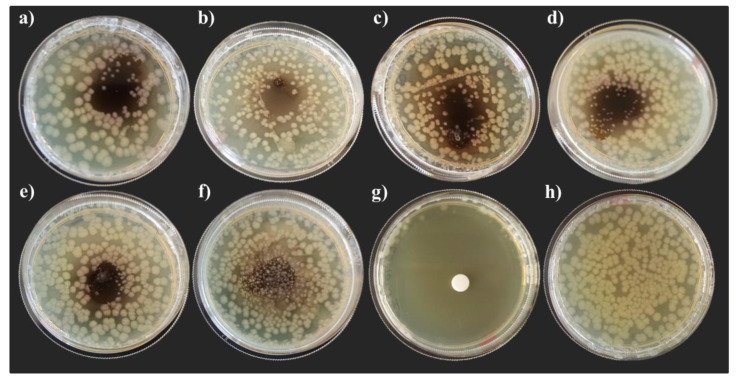
Growth inhibition of *E. coli* DH5α after 48 h of incubation. Radial diffusion assay: (**a**) hemolymph of African prepupae reared on NatOvo, (**b**) hemolymph of BEF Biosystems prepupae reared on NatOvo, (**c**) hemolymph of BEF Biosystems prepupae reared on FV, (**d**) hemolymph of BEF Biosystems prepupae reared on FVB, (**e**) hemolymph of BEF Biosystems prepupae reared on FVBD, (**f**) hemolymph of BEF Biosystems prepupae reared on FVBM, (**g**) antibiotic-treated control, (**h**) untreated control.

After 48 h of incubation (Figure 3), the inhibitory activity was still evident in all theses with the exception of the plates inoculated with hemolymph of the BEF Biosystems prepupae reared on FVBM (Figure 3f). Moreover, after 48 h of incubation, bacterial colonies deriving from the hemolymph itself were observed in plates inoculated with hemolymph of the BEF Biosystems prepupae reared on FVBD and FVBM (Figure 3f).

#### 3.3.2. *Micrococcus yunnanensis* HI55

**Figure 4:**
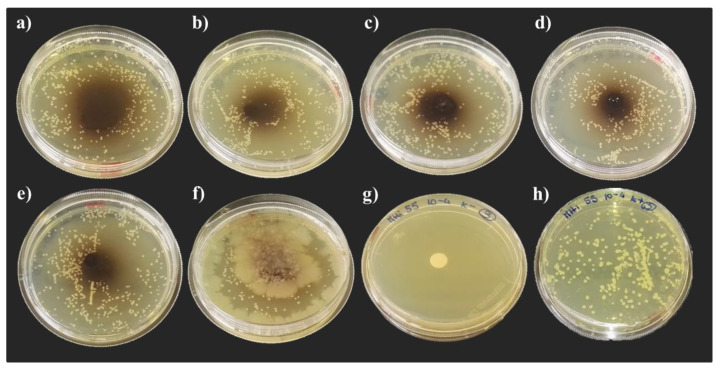
Growth inhibition of *M. yunnanensis* HI55 after 24 h of incubation. Radial diffusion assay: (**a**) hemolymph of African prepupae reared on NatOvo, (**b**) hemolymph of BEF Biosystems prepupae reared on NatOvo, (**c**) hemolymph of BEF Biosystems prepupae reared on FV, (**d**) hemolymph of BEF Biosystems prepupae reared on FVB, (**e**) hemolymph of BEF Biosystems prepupae reared on FVBD, (**f**) hemolymph of BEF Biosystems prepupae reared on FVBM, (**g**) antibiotic-treated control, (**h**) untreated control.

After 24 h, the inhibitory activity against *M. yunnanensis* HI55 was observed in all theses, except when the hemolymph of insects reared on the FVBM was used (Figure 4). For the NatOvo diet, a higher inhibitory activity was observed with hemolymph extracted from African prepupae compared to the BEF Biosystems insects’ hemolymph. Indeed, no colonies were observed in the plates inoculated with hemolymph extracted from African prepupae (Figure 4a). Similar inhibitory activities were observed among the FV and FVB theses (Figure 4c,d). No bacteria colonies were recorded in the area treated with hemolymph extracted from insects fed an FVBD diet (Figure 4e). After 48 h of incubation, the inhibitory activity was still evident (Figure 4f).

**Figure 5:**
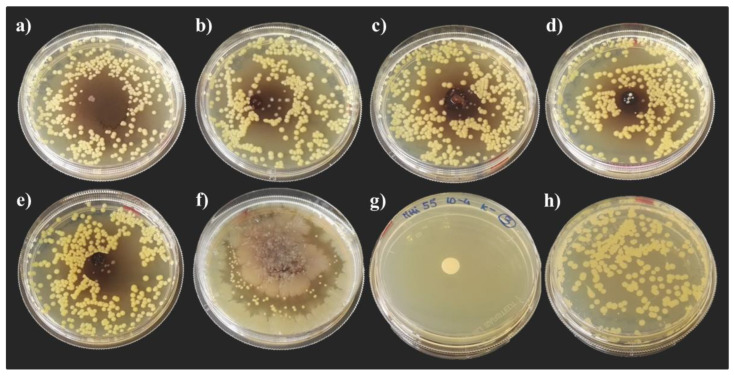
Growth inhibition of *M. yunnanensis* HI55 after 48 h of incubation. Radial diffusion assay: (**a**) hemolymph of African prepupae reared on NatOvo, (**b**) hemolymph of BEF Biosystems prepupae reared on NatOvo, (**c**) hemolymph of BEF Biosystems prepupae reared on FV, (**d**) hemolymph of BEF Biosystems prepupae reared on FVB, (**e**) hemolymph of BEF Biosystems prepupae reared on FVBD, (**f**) hemolymph of BEF Biosystems prepupae reared on FVBM, (**g**) antibiotic-treated control, (**h**) untreated control.

Moreover, in the area treated with hemolymph, the development of bacterial colonies originating from the hemolymph itself was observed. Indeed, in the area treated with the hemolymph of prepupae fed an FV and FVB, white bacterial colonies were already present after 24 h of incubation (Figure 4c,d) and a well distributed white bacterial film was observed in all plates inoculated with hemolymph extracted from insects reared on an FVBM diet (Figure 4f). After 48 h of incubation (Figure 5), colonies originating from the hemolymph itself were detected also in the area treated with the hemolymph of prepupae fed a NatOvo diet (both populations, Figure 5a,b) and fed an FVBD diet (Figure 5d).

## 4. Discussion

The black soldier fly larvae have been proposed as a promising solution to recycle food waste thanks to their ability to feed on a wide range of organic substrates, characterized by a different nutrient content. To date, in Europe, the use of catering waste (Reg. (EC) No. 1069/2009) and food by-products containing meat and fish (Reg. (EU) No. 142/2011) as rearing substrates is still facing legal obstacles for insects intended as food or feed. Different combinations of fruit and vegetable-based diets have been proposed. This is because fruit and vegetable waste represent the highest proportion of food waste and loss mostly due to postharvest grading, food industry processing and retail and consumers’ garbage [38]. However, prior to being used for large-scale production, food waste-based substrates should be carefully evaluated. Concerning safety aspects, attention has to be paid to a potential contamination of the rearing substrate with heavy metals and mycotoxins and their transfer to and possible accumulation in insects. Moreover, these diets could be nutritionally unbalanced and even have deficiencies that could lead to a weakening of the immune system itself. For insect mass-rearing facilities, it is important to maximize insect production and guarantee insect health. Therefore, assessing the effect of different food waste-based diets on both the insects’ growing performances and their immunity is essential.

In this study, we investigated the growing performances, waste reduction and immune responses of BSF larvae reared on different catering waste. Larval final weight and ADG were positively affected by the NatOvo diet, as already reported by Bava et al. [39], and by the FVBM diet. Indeed, as previously observed [40,41], a higher growth rate was recorded in insects fed a protein-rich diet that, being more balanced, provided the macro- and micronutrients necessary for insect development [42,43,44]. A higher meat inclusion rate in the diet (80%), compared to the one used in our trials (10%), led to an extreme larval weight and survival reduction due to excessive protein and fat intake [45]. The lowest larval final weight and ADG observed in the FV diet are probably due to the lower protein content of the diet itself [46]. Moreover, it has to be taken into account that, in our trial, ADG was only evaluated after 7 days of permanence on the tested diet, and this parameter could increase significantly over the time as already reported for BSF larvae reared on an FV diet [46]. Although the FVB diet is not particularly rich in protein, similar ADG values have been observed in insects fed an FVB and an FVBD diet. The addition of dairy products in the diet (FVBD) did not result in larval weight and ADG similar to the ones observed in the diet supplemented with meat (FVBM), probably due to lower nutritional content (e.g., vitamin B_12_ content).

The survival rate was high across all tested diets ranging from 75 to 97%. A lower survival rate has been recorded in insects fed a NatOvo and an FVBM diet, while the FV diet allowed the highest survival rate. As previously discussed, a fruit and vegetable-based diet, with the lowest protein content, negatively affects the growth rate, ADG and final production of larval biomass, but at the same time ensures higher larval survival [45,46]. A higher final production of larval biomass was observed when feeding the insects a NatOvo, FVBM, and FVBD diet, respectively. In our trials, the substrate reduction ranged from 64 to 86%, similarly to what was obtained in other trials were BSF larvae reduced the substrate biomass by more than 50% [39,47]. In our trial, the NatOvo diet led to the highest substrate reduction, followed by the FVB and FVBM diets. At the moment, food waste containing meat or fish are not allowed in animals’ diet by the European legislation, and it is not easy, under a practical point of view, to segregate catering waste in order to prevent food by-products containing meat and fish. Therefore, one of the current goals of European insect producers and the International Platform of Insects for Food and Feed (IPIFF) is the diversification of the spectrum of substrates allowed in insect farming, by including former foodstuffs containing meat and fish and subsequently catering waste to the list of authorized substrates. This will enhance the circularity of insect production, thus helping European insect farms to reach their full potential. The inclusion of former foodstuffs containing meat and fish, followed by catering waste, will be an essential pillar: such materials, not suitable for other farmed animals, are better upcycled by insect bioconversion [48]. Moreover, good organization in the collection of local catering waste could be a key step for the development of a feedstock supply chain for BSF larvae production [49]. Our results provide important background knowledge on the use of food residues also containing meat or fish, with a view to achieving the crucial goal described above.

Although there is an increasing amount of research aimed at evaluating the growth performance of BSF reared on catering waste and municipal organic waste, the influence that such diets may have on the insect’s immune system is still only marginally considered. Nutrient-poor diets and non-optimal protein–carbohydrate ratios result in a lower production of AMPs and hemocytes and a decrease in the insect endogenous bacteria [25,50,51]. The effect of the diet on insect immune responses has been highlighted [27,28,29,30,31], but the diet-mediated effect on both growing performance and immunity is still poorly investigated both in useful and pest insects. For example, the preference of the cabbage moth *Plutella xylostella* (L.) (Lepidoptera: Plutellidae) for the mustard plant is well known, despite its relatively poor performance on this plant. Only recently, the possible reason for this choice has been described. It seems to be related to the major hemocyte counts and melanization capacity obtained when caterpillars fed on mustard compared to other plants, suggesting that protection from parasitoids and entomopathogens is more important than improving the growth rate [52]. Moreover, the growth level of *H. cunea* reared on different host plants did not predict the insect’s immune responses [32]. Therefore, assessing the impact of the diet on insect immune responses could highlight the need to supplement with probiotics or similar products (that act positively on the immune system) all those diets based on food waste that are not favorable to the immune system or are less favorable compared to others. Insects produce the most diverse AMPs for defence, and BSF is one of the most promising sources for AMPs. In addition to their antimicrobial effect, AMPs positively affect growth and immune responses, including increased immunity in pigs [53], improved chicken growth and meat quality, and increased fish growth rate [54].

The possibility to modulate AMP production with the diet opens new perspectives in the use of BSF as feed not only in terms of nutritional values but also as a novel feed ingredient that can improve the health of aquaculture products with immunity-enhancing effects. In particular, a diet-dependent expression of AMPs translated into diet-dependent profiles of inhibitory activities against a spectrum of bacteria opens a new and important chance to transfer these AMPs in the animals fed with BSF larvae. In our trials, AMP-encoding gene expression levels were differently modulated depending on the insect population and the rearing diet. A slight down-regulation of the defensin (fold change 0.97) and an up-regulation of the cecropin-encoding gene (fold change: 1.62) were observed in insects that originated from Africa compared to insects reared at the BEF Biosystems (both fed the NatOvo diet). In our trials, the F2 generation of the African population has been used in a preliminary test in order to evaluate the possible differences in AMPs’ modulation also due to genetic factors. Indeed, the results we obtained are not sufficient to state that AMP transcription levels are higher in this population, but they underline a possible influence of genetic factors that may be useful in insect breeding. For example, in wild honeybee colonies, genetic diversity is positively associated with immunocompetence. It has been hypothesized that wild populations could be useful sources of genetic variation to be used in breeding programs to improve honeybee health [55]. Similarly, BSF populations with higher immune responses mediated by genetic factors could be used in breeding programs.

In our trials, the higher overexpression of both AMP-encoding genes was observed in prepupae fed all tested diets, with the exception of insects fed an FV diet. In this last case indeed, a down-regulation of both AMP coding genes was observed, probably due to the lower nutrient composition [25] compared to the control diet. Higher expression levels both for defensin and cecropin were recorded for the FVB and FVBD diets, while only defensin was significantly up-regulated in insects fed an FVBM diet. Both AMPs’ transcripts were significantly up-regulated in prepupae fed a dairy product-added diet showing a 2-fold upper transcript level as compared with insects fed an FVBM, and 5-fold and a 2-fold upper transcript levels when compared to an FVB diet for defensin and cecropin, respectively. As previously shown, a higher microbial load of the substrate may lead to the major expression of AMP-encoding genes [56,57], and the bacteria present in the dairy products may act as probiotics.

The possible role of dairy products in the diet of BSF larvae requires further investigation on the reproductive performances. Indeed, unexpectedly, the presence of fermented milk and dairy products (yogurt and kefir) in the diet of the fruit fly, *Drosophila melanogaster* Meigen (Diptera: Drosophilidae), negatively affected both the larval development and eggs laid [58]. It has been suggested that the dairy products may alter the pH of the diet influencing the development of acetic bacteria, the major component of *D. melanogaster* microbiota, or they could limit the development of yeasts, the main protein source for this insect, with negative consequences on larval growth [58]. Similar results were recorded also when milk was added to the housefly, *Musca domestica* L. (Diptera: Muscidae), with the adult diet affecting both daily egg production and egg weight [59].

In our trials, we only investigated the diet-dependent expression level of two AMPs. However, it is important to highlight that the tested diets may have also influenced the transcription of other AMPs that could also be involved in the hemolymph inhibitory assay. The hemolymph is well-defended by hemocytes and by various soluble molecules with antimicrobial functions [60] including AMPs, thio-ester proteins as well as the prophenoloxidase cascade products [61,62,63]. The inhibitory activity against bacterial colonies observed in our trials was only due to the different humoral immune responses and to their synergic action because we immediately discarded hemocytes after the hemolymph collection, by precipitation. In our trials, the hemolymph inhibitory activity was evident. A reduction in the number and size of *E. coli* DH5α and *M. yunnanensis* HI55 bacterial colonies was observed following the addition of the hemolymph extracted from individuals (African and BEF Biosystems origin) reared on the different tested diets. While in other trials, the inhibition activity against Gram-negative bacteria was observed to persist only for 24 h [56], in our study the hemolymph inhibitor activity against both Gram types was still present after 48 h of incubation. Moreover, in our trial, the inhibition zone was determined only by the diet and not following the inoculation of insects with entomopathogens as reported in other studies [64,65,66,67].

In addition, we observed the growing of morphologically different bacterial colonies deriving from the hemolymph itself extracted from insects fed different substrates. The development of these microorganisms was evident in all plates treated with hemolymph and inoculated with *M. yunnanensis* HI55; however, their growth was particularly evident and widespread in plates treated with the hemolymph of prepupae reared on the FVBM diet. In contrast, in plates inoculated with *E. coli*, colonies were observed only where the hemolymph of prepupae reared on the FVBM diet was added. Healthy insect hemoplymph has long been considered a hostile environment for microorganisms, and therefore microbiologically sterile [68]. There is now evidence that various non-pathogenic microorganisms stably or transiently inhabit hemolymph in a diversity of insects [60], and that hemolymph microbiota could positively affect the immune responses.

The hemolymph microbiota–insect host interactions, as well as the function of hemolymph microbiota, are still unclear, and further research is needed in order to deeply investigate these aspects. The identification of the microorganisms developed from the hemolymph could offer new perspectives in order to better understand the relation between the hemolymph microbiota, the diet and the insect’s immune system. It would be also interesting to assess whether hemolymph microbiota may up-regulate the immunity-related genes, containing pathogen recognition receptors and AMPs, as already reported for the red palm weevil, *Rhynchophorus ferrugineus* Olivier (Coleoptera: Dryophthoridae), gut microbiota [69] and mosquito [70,71,72]. Moreover, further investigations are required in order to clarify whether the hemolymph microbiota can have an active role against entomopathogens, not only due to space and nutrition competitions.

## 5. Conclusions

Nowadays, there is an urgent need to reduce waste in food production and find solutions for food waste recycling and valorization. The insect-based bioconversion represents an economically viable solution for food waste management. For insect mass-rearing facilities, it is important to maximize insect production and guarantee insect health. Therefore, assessing the effect of different food waste-based diets on both insects’ growing performances and their immunity is essential. Indeed, these diets could be nutritionally unbalanced and even have deficiencies that could lead to a weakening of the immune system itself. Evaluating the impact of the diet on insect immune responses could highlight the need to supplement with probiotics or similar products (that act positively on the immune system) all those diets based on food waste that are not favorable to the immune system or are less favorable compared to others. In our trials, the dairy product-added diet (FVBD) let to the higher expression of both AMP-encoding genes. However, the possible role of dairy products in the diet of BSF requires further investigation, considering their negative impact on larval growth and the reproductive performance of other Diptera species. In this scenario, our results open up new perspectives for the optimal utilization of different food waste, especially if the European legislature will extend the spectrum of allowed substrates to former foodstuffs containing meat and fish. This will enhance the circularity of insect production, thus helping European insect farms to reach their full potential.

## Figures and Tables

**Table 1:** Value (mean ± SE) of the final weight of the single larva, ADG, survival rate, final larval biomass and substrate reduction obtained by rearing the black soldier fly (*Hermetia illucens*) larvae on different catering waste-based diets for 7 days. For each parameter, means followed by different letters are significantly different (one-way ANOVA: final weight, ADG and final larval biomass; Kruskal–Wallis test: survival rate and substrate reduction; *p* ≤ 0.05).

Parameter	NatOvo	FV	FVB	FVBD	FVBM
Final weight (mg)	181.10 ± 5.71 a	115.43 ± 3.10 c	148.18 ± 1.95 b	149.33 ± 2.33 b	176.80 ± 2.00 a
ADG (mg day^−1^)	23.01 ± 0.81 a	13.49 ± 0.44 c	18.17 ± 0.28 b	18.33 ± 0.33 b	22.26 ± 0.29 a
Survival rate (%)	75.54 ± 2.67 d	97.19 ± 0.31 a	82.44 ± 0.29 c	88.11 ± 1.07 b	76.80 ± 1.25 d
Final larval biomass (mg)	274.50 ± 1.18 a	224.40 ± 6.50 c	244.30 ± 2.44 b	263.07 ± 2.00 a	271.47 ± 1.75 a
Substrate reduction (%)	86.33 ± 0.38 a	64.33 ± 2.60 d	76.90 ± 0.38 b	73.87 ± 0.26 c	76.10 ± 0.12 b

## Data Availability

Not applicable.
